# Cost analysis of a school-based comprehensive malaria program in primary schools in Sikasso region, Mali

**DOI:** 10.1186/s12889-017-4490-6

**Published:** 2017-06-12

**Authors:** Roberta Maccario, Saba Rouhani, Tom Drake, Annie Nagy, Modibo Bamadio, Seybou Diarra, Souleymane Djanken, Natalie Roschnik, Siân E. Clarke, Moussa Sacko, Simon Brooker, Josselin Thuilliez

**Affiliations:** 1 Department of Social Policy, London School of Economics, London, UK; 2 London School of Hygiene and Tropical Medicine, London, UK; 3 Save the Children, Bamako, Mali; 4 Institut National de Recherche en Santé Publique, Bamako, Mali; 5 CES-CNRS, Université Paris 1, Panthéon-Sorbonne, Centre d’économie de la Sorbonne, Maison des Sciences Economiques, 106-112 Boulevard de l’Hôpital, 75013 Paris, France

**Keywords:** Malaria, Cost analysis, Malaria control, Schools, School health, LLINs, Intermittent treatment, IPCs, IPT, Programme costs, Mali

## Abstract

**Background:**

The expansion of malaria prevention and control to school-aged children is receiving increasing attention, but there are still limited data on the costs of intervention. This paper analyses the costs of a comprehensive school-based intervention strategy, delivered by teachers, that included participatory malaria educational activities, distribution of long lasting insecticide-treated nets (LLIN), and Intermittent Parasite Clearance in schools (IPCs) in southern Mali.

**Methods:**

Costs were collected alongside a randomised controlled trial conducted in 80 primary schools in Sikasso Region in Mali in 2010-2012. Cost data were compiled between November 2011 and March 2012 for the 40 intervention schools (6413 children). A provider perspective was adopted. Using an ingredients approach, costs were classified by cost category and by activity. Total costs and cost per child were estimated for the actual intervention, as well as for a simpler version of the programme more suited for scale-up by the government. Univariate sensitivity analysis was performed.

**Results:**

The economic cost of the comprehensive intervention was estimated to $10.38 per child (financial cost $8.41) with malaria education, LLIN distribution and IPCs costing $2.13 (20.5%), $5.53 (53.3%) and $2.72 (26.2%) per child respectively. Human resources were found to be the key cost driver, and training costs were the greatest contributor to overall programme costs. Sensitivity analysis showed that an adapted intervention delivering one LLIN instead of two would lower the economic cost to $8.66 per child; and that excluding LLIN distribution in schools altogether, for example in settings where malaria control already includes universal distribution of LLINs at community-level, would reduce costs to $4.89 per child.

**Conclusions:**

A comprehensive school-based control strategy may be a feasible and affordable way to address the burden of malaria among schoolchildren in the Sahel.

**Electronic supplementary material:**

The online version of this article (doi:10.1186/s12889-017-4490-6) contains supplementary material, which is available to authorized users.

## Background

The vast majority of malaria morbidity and mortality occurs in sub-Saharan Africa, where infection with *Plasmodium falciparum* remains a considerable obstacle to both health and economic development. Traditionally, efforts have targeted maternal and child morbidity and mortality driven by the persistent malaria burden during pregnancy and early childhood. However, an increasing body of evidence points to broader social and economic impacts of the disease [1–7]. In particular, there is increased recognition of the value of including of school-aged children in these efforts, due to the high parasite rates in this population and the increasing body of evidence demonstrating developmental consequences of infection throughout childhood and adolescence [8, 9].

Malaria has been associated with reductions in sustained attention, cognition, and school achievement in various settings, including Mali, where this study is based. Analysis of data from demographic and health surveys found that a higher prevalence of malaria in a community was associated with higher primary repetition rates [3]. In addition, a longitudinal survey in an area with high and stable malaria transmission area has shown that *Plasmodium falciparum* infection affects educational achievement and cognitive performance [4]. Similar results have been observed elsewhere [5–7].

School-based delivery of interventions represents a potential strategy to address this burden. A cluster-randomised trial in western Kenya demonstrated schools as a promising and cost-effective channel through which to deliver malaria treatment to schoolchildren and promote improvements in attention and school performance [6, 10]. There are few data on the cost or cost-effectiveness of school-based interventions. A systematic review in 2011 identified 48 studies that evaluated the cost-effectiveness of malaria interventions [11], of which only two [10, 12], were conducted among school-aged populations; as well as a cost analysis of a more recent school-based intervention in Kenya [13]. Better understanding the financial and economic costs of school-based interventions will help to inform ongoing questions of whether malaria control programmes can be channelled through schools, particularly in light of the availability of new preventive strategies such as seasonal malaria chemoprevention [14, 15], and whether integrated programmes rather than single interventions should be implemented [10, 13, 16].

The main objective of this study is to describe the costs of a comprehensive school-based malaria intervention strategy carried out in an area of highly seasonal malaria transmission in Mali, following an ingredients approach. The intervention included three components: participatory malaria education; distribution of long-lasting insecticide-treated nets (LLIN); and a 3-day course of treatment with artemisinin-combination therapy administered at the end of the transmission season to all schoolchildren (intermittent parasite clearance in schools, or IPCs). Key cost drivers are examined to inform a discussion of factors to consider in implementing or scaling up the programme at the national level.

## Methods

### Intervention and study site

The cost analysis was part of a cluster-randomised trial of a school-based malaria intervention carried out by Save the Children International (STC) in 80 primary schools in Sikasso Region in south-eastern Mali between November 2010 and March 2012. The impact of the intervention on health and education outcomes is reported elsewhere [17]; including details of the study site. In brief, Sikasso is characterized by highly seasonal malaria transmission which occurs during the single rainy season between May and November each year. Schools were eligible for inclusion in the trial if they had at least 50 students and taught grades 4 and 5, and were randomized to either the intervention arm or the control arm. The overall purpose of the study was to evaluate the impact of a comprehensive malaria intervention on health and school performance, measuring clinical indicators such as malaria infections and anaemia, as well as cognitive outcomes, such as sustained attention.

### Comprehensive malaria intervention in schools

The intervention included three components: (i) participatory malaria prevention education; (ii) distribution of LLINs before the start of the rainy season; and (iii) administration of a 3-day antimalarial treatment to all schoolchildren to eliminate any malaria infections at the end of the malaria transmission season (intermittent parasite clearance in schools, or IPCs). All three components of the intervention were delivered by teachers.

**Malaria education** start-up costs included a preliminary phase to develop the health education guidance manual on malaria prevention and effective use of LLIN, including examples of educational activities that could be carried out in schools, to be utilized by teachers, followed by a two-day teacher training workshop carried out by Save the Children personnel (from January to April 2011). A total number of 177 teachers from the 40-targeted schools were trained for 2 days. After training, teachers were given 2 weeks to administer lessons, lead participatory learning activities with pupils and organize a ‘malaria awareness day’, at which students would perform dramas or songs to raise awareness in their local community.

**LLIN distribution** occurred at schools during the ‘malaria awareness day’ in April 2011. Each schoolchild was given two nets to ensure the availability of nets in the home for their use. Long lasting insecticidal nets (LLIN) are manufactured with an insecticide incorporated into the net fabric which makes the insecticide last for at least 20 washes in standard laboratory testing, or approximately 3 years of use under field conditions; and widely promoted by WHO and Roll Back Malaria partners as a cost effective and sustainable method for protection against malaria.

**IPCs administration** consisted of a single annual treatment at the end of the transmission season (in November 2011), given to all children attending primary school in classes 1-6. A training workshop was held to instruct teachers, health officers and directors of community health programme (DTCs) on how to administer the drugs to children, dosage to use, and potential side effects. Treatment with the selected drug, Sulfadoxine and Pyrimethamine (SP) and Artesunate (AS commercial name *Artecospe*®), was given according to age. Each child received treatment at the school for three consecutive days. Children absent on the first day were excluded from the treatment; those who received treatment on day 1 but were absent thereafter were traced and treated at home. Teachers were responsible for calculating the amount of tablets for their classes, administering the treatment and keeping records. STC personnel were present in each school on the first day only.

**Children in control schools** received LLINs through a Malian government implemented universal distribution of LLINs targeting the general population in Sikasso Region in April 2011, giving one LLIN per two persons in each family. Costs of this government intervention were inaccessible for the purposes of this analysis. Although these children had LLINs during the trial period, they did not receive malaria education in schools to promote their use, or IPCs at the end of the transmission season. After the end of the trial, the full intervention was rolled out to schools in the control group in March 2012.

### Costing

The analysis has been conducted from a service provider perspective [18]. However, costs to recipients are likely to be negligible since the intervention was delivered directly through school and without user fees of any kind [13]. The time horizon of the analysis is 1 year, with costs presented for the delivery of one annual round of the entire intervention.

Cost data were collected in situ by STC personnel based in Mali between November 2011 and March 2012, using worksheets created specifically for the cost analysis. Except for the drugs, whose costs were in United States Dollars (USD), all other costs were collected in local currency West African Francs (XOF). Results of the cost analysis are expressed in XOF and USD 2012 (average exchange rate, first quarter 2012; 1 USD = 510.49 XOF) [19].

Based on general principles of cost analysis and specific recommendations for malaria projects [20–22], the study followed an ingredients-based approach, meaning that the different resources necessary to carry out the intervention were listed, measured and valued (Additional file 1). Costs were then classified according to activity as illustrated in Table 1. One advantage of this approach is that costs can be analysed in detail and cost drivers can be identified. Additionally, starting from the base case intervention, different scenarios can easily be drawn through changes in ingredient quantities and values: this is particularly useful in order to adjust the intervention for possible replications and for comparison with other studies [21].

**Table 1:** Activities of the comprehensive malaria control intervention carried out in primary schools in Sikasso, Mali

1	Planning and management	Refers to STC role in implementing and facilitating the intervention along the different operational phases
2	Developing of teaching materials	Includes the preparation of a manual for teachers, adapting material used in other countries
3	Community sensitization	Refers to involving parents and the whole community in educational activities about using LLIN and in organizing a ‘bed net distribution day’
4	Training	Training of trainers and training of teachers were carried out about malaria prevention, correct use of LLIN and IPCs administration. Directors of community health programmes participated as well
5	Lessons for children	Concerns the time spent by teachers in preparing and conducting lessons in school
6	Purchase, transport and storage	STC organized the purchase of LLIN (locally) and drugs (imported) and the delivery to the designed villages
7	Distribution, dispensing	Refers to the actual distribution of LLIN and dispensing of drugs to the beneficiaries

*STC* Save the Children, *LLIN* long-lasting insecticidal net, *IPCs* intermittent preventive treatment in schools

A standard template for cost analysis on malaria projects is not available [21]. For the purpose of this study, costs classified under start-up costs refer to those costs incurred for initiating the programme, and operational costs (post start-up) refer to the costs of delivering the intervention to the beneficiaries. Costs were next attributed to the following categories (i) Human resources (including salaries and allowances provided to STC personnel and teachers), (ii) Materials (including consumables for training), (iii) Logistics and transport (including costs for custom, transport and storage of intervention items and communication means), (iv) Intervention items (costs of LLIN and drugs). Joint costs, such as STC personnel, vehicle, storage and office utilities, have been allocated to the intervention according with their usage [23]. LLIN were considered to be capital items with 3-year life span [21]. Annual financial costs were calculated with straight-line depreciation, whereas for economic costs discount rate of 3% was considered to calculate the relevant annualization factor [24]. We calculated both financial and economic costs, the latter including the opportunity costs of utilizing resources such as training facilities provided by the community and teachers’ time, since activities were carried out during their regular working hours and rewarded through government salary.

### Sensitivity analysis

**Fig. 1:**
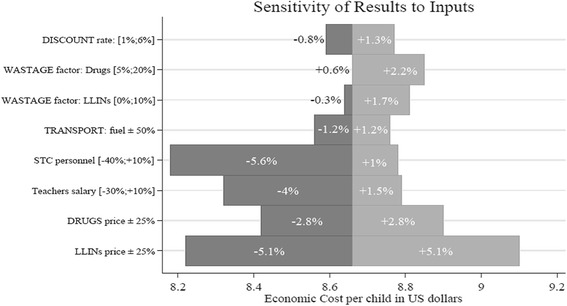
Sensitivity analysis (based on ‘adapted intervention’). Lower sensitivity values are provided in dark grey. Higher sensitivity values are provided in light grey

We analysed the impact of cost variations and the level of uncertainty associated with relevant parameters used in the cost estimation with a univariate sensitivity analysis. Analysis was performed on LLIN prices (± 25%), drugs prices (±25%), salary levels including school teachers salaries (−30%,+10%), STC personnel (−40%,+10%) and transport costs (±50%). Additionally, variation in wastage factor for LLIN (0%,20%) and drugs (5%,20%) and discount rate (1%,6%) were analysed. Results are displayed graphically using a Tornado diagram (Fig. 1). For intervention items, salary and transport costs the range of variations were chosen to reflect market price variations, based on expert opinion and realistic range of changes. For wastage factor, values were based both on literature [10, 13] and expert opinion. Finally, the choice of range values for discount rate follows Kolaczinski and Hanson recommendation [21].

To provide meaningful comparisons for consideration of program replication or adoption, we also evaluate the cost of two adapted intervention designs. These adaptations were informed by consultation with implementation partners involved in the trial who identified modifications to the strategy that might limit resource expenditure and improve intervention coverage and impact; and entail inclusion of all children in the IPCs component, irrespective of any absences on the first day of treatment, and delivering fewer LLIN to each child.

### Ethical clearance

Local approval for the intervention was granted by the Ethical Committee of the National Institute of Research in Public Health, Ministry of Health of the Republic of Mali (00013/10/CE-INRSP). Approval for the cost analysis was received from LSHTM Research Ethics Committee [reference number 011267]. STC was responsible for implementation of project activities including, procurement of supplies, technical support, training and supervision.

## Results

### Actual intervention

The number of children targeted in the intervention was 6413, with an actual coverage of 96.7% for the LLIN distribution and 96.0% for completion of the 3-day IPCs treatment regimen.

#### Total costs

Total financial costs for the intervention as implemented were estimated to be 53,927 USD (27,529,601 XOF), with an economic cost of 66,554 USD (33,975,552 XOF). Key differences between financial and economic costs are due to the opportunity cost of teachers’ time. Delivering malaria lessons took on average three full days, and administering one IPC dose required an average of 1 min per child. Table 2 provides details of the results with a breakdown of the costs for the three components of the intervention. The LLIN intervention accounted for the largest portion of the total costs (58% of financial costs, 53% of economic costs), while the IPCs component accounted for approximately one quarter (27% of financial costs, 26% of economic costs). For a more accurate understanding of the intervention costs, Table 3 presents costs divided per categories and per activities. Our results suggest that human resources were the major driver of costs, followed by LLIN procurement. Similarly, the activity breakdown shows that LLIN purchase, transport and storage costs counted for more than one third of total costs. Training activities and resources for 8 trainers, 177 teachers and 17 supervisors represented the same proportion of the total cost.

**Table 2:** Financial and economic costs of a comprehensive malaria intervention in schools in Sikasso region – Mali: Actual intervention

	Actual intervention							
	Complete intervention		Component 1: Malaria education		Component 2: LLIN distribution		Component 3: IPCs distribution	
	Financial cost	Economic cost	Financial cost	Economic cost	Financial cost	Economic cost	Financial cost	Economic cost
Total costs								
in XOF	27,529,601	33,975,552	4,088,728	6,969,781	15,906,552	18,106,176	7,534,321	8,899,595
in USD	53,927	66,554	8009	13,653	31,159	35,468	14,759	17,433
Cost per child								
in XOF	4293	5298	638	1087	2480	2823	1175	1388
in USD	8.41	10.38	1.25	2.13	4.86	5.53	2.30	2.72

**Table 3:** Detailed financial and economic costs of comprehensive malaria intervention in schools in Sikasso – Mali: Actual intervention

	Actual intervention					
	Financial cost (in XOF)	Financial cost (in USD)	Cost profile	Economic cost (in XOF)	Economic cost (in USD)	Cost profile
	By cost category					
Start-up costs^a^						
Human Resources	9,012,403	17,654	33%	11,635,846	22,793	34%
Materials	1,321,640	2589	5%	1,321,640	2589	4%
Logistics and transport	20,000	39	0.1%	20,000	39	0.10%
Operational costs (post start-up)						
Human resources	2,264,530	4436	8%	5,288,189	10,359	16%
Logistics and transport	922,942	1808	3%	1,064,414	2085	3%
Intervention items						
LLINs	10,848,658	21,251	39%	11,506,037	22,539	34%
Drugs	3,139,427	6150	11%	3,139,427	6150	9%
	By activity					
Planning and management	2,149,871	4211	8%	2,461,362	4822	7%
Developing teaching material	343,203	672	1%	412,424	808	1%
Community sensitization	32,427	64	0.1%	1,368,375	2680	4%
Training	9,416,960	18,447	34%	12,091,183	23,685	36%
Lessons for children	0	-	0%	584,477	1145	2%
LLIN purchase, transport and storage	11,553,856	22,633	42%	12,221,970	23,941	36%
LLIN distribution	367,640	720	1%	800,081	1567	2%
Drugs purchase, transport and storage	3,665,643	7181	13%	3,835,798	7514	11%
Drugs dispensing	0	-	0%	199,883	392	1%
Total	27,529,601	53,927	100%	33,975,552	66,554	100%

^a^Includes costs of training (training of trainers and training of teachers) for 8 trainers and 117 teachers in 40 schools

#### Cost per child

The financial costs to provide the comprehensive intervention to one child are estimated at 8.41 USD (4293 XOF), whereas economic costs are 10.38 USD (5298 XOF) per child. These figures may be considered as cost per child per year, assuming that IPCs is delivered annually. However, it is worth noting that the annualized LLIN cost may be somewhat inflated due to the fact that it is not necessary to transport and distribute new LLINs to all children every year. Results provided by Table 2 assume that there are 160 pupils in each school, reflecting the mean school size observed among schools participating in the trial (range 50-517 pupils). Although variation in cost per child in relation to school enrolment will not be further elaborated in this study, they might be relevant for budget impact analysis in school at local level.

### Adapted intervention scenarios

Adapted implementation scenarios are also considered based on likely or feasible programmatic changes. Details of results, financial and economic costs of these adapted intervention scenarios are summarized in Table 4. In adaptation 1: two modifications were considered based on consultation with implementation partners involved in the trial who highlighted these as potential strategies to limit resource expenditure and improve intervention coverage and impact. The first modification considered was to distribute only one LLIN per child. Compared to the actual intervention, in which each child received two nets, this adjustment generates a significant reduction of cost for the activity ‘LLIN purchase, transport and storage’, from 23,941 USD to 12,659 USD economic costs (Table 3, Additional file 2). Second, the IPCs component should aim to cover the highest possible number of children and, once the treatment is initiated, increase efforts to complete it. Children who are absent on day 1 would therefore be included in the intervention that would then continue on day 4. The calculation of these additional costs is based on the percentage of absenteeism observed (2.60%, 3.25%, 4.05% on day 1, 2, 3 respectively). In terms of impact on both financial and economic costs of the IPCs component, the difference between actual and adapted intervention is minimal (increase of 0.04 USD per child). Total economic costs under the adapted model 1 are estimated at 55,550 USD (8.66 USD per child), as opposed to 66,554 USD (10.38 USD) for the actual intervention. The overall cost reduction is 17%; the LLIN component on its own registers a decrease of 32%. After these adjustments, human resources remain the major cost driver in terms of cost categories, whereas training becomes the driver among the activities.

**Table 4:** Financial and economic costs of a comprehensive malaria intervention strategy for use in schools in areas of seasonal transmission: Adapted versions for future scale-up

Adapted Intervention 1: For use in settings with LLINs distribution targeted to pregnant women and children under-five years								
	Complete Intervention		Component 1: Malaria Education		Component 2: LLIN Distribution^a^		Component 3: IPCs Distribution	
	Financial Cost	Economic Cost	Financial Cost	Economic Cost	Financial Cost	Economic Cost	Financial Cost	Economic Cost
Total Costs								
in XOF	22,238,891	28,357,759	4,088,728	6,969,781	10,475,398	12,346,332	7,674,765	9,041,646
in USD	43,563	55,550	8009	13,653	20,520	24,185	15,034	17,712
Cost per Child								
in XOF	3468	4422	638	1087	1633	1925	1197	1410
in USD	6.79	8.66	1.25	2.13	3.20	3.77	2.34	2.76
Adapted Intervention 2: For use in settings with regular universal distribution of LLINs targeting all age groups								
	Complete Intervention		Component 1: Malaria Education		Component 2: LLIN Distribution^b^		Component 3: IPCs Distribution	
	Financial Cost	Economic Cost	Financial Cost	Economic Cost	Financial Cost	Economic Cost	Financial Cost	Economic Cost
Total Costs								
in XOF	11,763,493	16,011,427	4,088,728	6,969,781	0	0	7,674,765	9,041,646
in USD	23,043	31,365	8009	13,653	0	0	15,034	17,712
Cost per Child								
in XOF	1835	2497	638	1087	0	0	1197	1410
in USD	3.59	4.89	1.25	2.13	0	0	2.34	2.76

^a^Assumes 1 LLIN distributed to each school child each year, compared to 2 LLINs per child in actual implementation.

^b^Assumes that all schoolchildren have previously received LLINs through universal distribution campaigns, and no additional LLIN distribution through schools is needed

In adaptation 2, a further modification entailed the removal of the LLIN component altogether. This adaptation is appropriate for settings where the national malaria control strategic plan includes universal net distributions targeting all age groups, and assumes that all schoolchildren would have previously received LLINs through a universal distribution campaign at community-level, and thus no additional LLIN distribution through schools is needed. Total economic costs under the adapted model 2 are estimated at 31,365 USD (4.89 USD per child); an overall cost reduction relative to the actual intervention of 47%.

### Sensitivity analysis

Figure 1 provides the details of the variables used in the sensitivity analysis, which is reported in terms of changes in economic costs. Intervention items were analysed first. It should be noted that during the programme implementation from November 2010 to March 2012, prices of LLIN, that were purchased locally, and of drugs, imported from China, were reported as stable. Nonetheless, an increase or decrease of 25% has been taken into account, considering possible future fluctuation in market prices. The impact on programme costs shows to be relevant, especially price increase for LLIN, which will result in total costs equal to USD 58,367 (+5.1%). The second element examined was variation in human resources costs. The current policy for government teachers does not foresee a regular salary adjustment to the cost of living; nevertheless, assuming a possible salary increase of +10%, total costs will increase to 56,362 USD and cost per child to 8.79 USD (+1.5%). This project was implemented by an international NGO, but for scale-up the training and logistic support would more likely be provided by staff employed by government or a local NGO. Regarding STC personnel, a reduction of −40% has therefore been envisaged considering that INGO’s remuneration level was higher than government pay scales. Impact on costs would then be important, resulting in a lower cost of 8.18 USD per child (−5.6%).An additional parameter considered was increase in fuel price, as per the current market trend. Since transport represents a minor cost component of the programme, +50% increase in fuel will be reflected in a cost per child of 8,76 USD (+1.2%). Finally, as suggested by the literature and other studies [10, 21, 23], the sensitivity analysis considered variation in discount rate and wastage factor. Differences due to discount rates are not significant. A wastage factor of 10% for LLIN would generate an increase in total costs up to 56,493 USD (+1.7%), similarly a high wastage factor for drugs, such as 20%, would result in total costs equal to 56,780 USD (+2.2%): this might occur in case of difficulties in managing timely procurement, item storage or distribution.

A final element worthy of consideration is the choice of drug for the IPCs component, following WHO recommendation in 2012 on use of Sulfadoxine/ Pyrimethamine and Amodiaquine (SP + AQ) for seasonal malaria chemoprevention in the Sahel sub-region [25]. In this case, drug price would decrease from 1.40 USD with SP/AS to 0.13 USD with SP/AQ for children of less than 7 years and from 0.85 USD to 0.25 USD for children older than 7 years [24]. Average cost per child would thus decrease to 7.91 USD (−9%).

## Discussion

Various factors must be considered in interpreting the costs of the school-based intervention described here. The first is the difference between a comprehensive intervention, as presented here, and other cost models for malaria control available in the literature which tend to focus on education, LLIN distribution, or treatment separately. Whereas in combined programmes cost savings and economies of scope can be generated by a joint delivery, the effectiveness of this programme in this area of seasonal malaria depends on the correct sequential timing of the activities: LLINs and malaria education are delivered ahead of the onset of the annual rains to protect schoolchildren from new infections during the period of highest risk when schools are closed, whereas IPCs is given several months later at the end of the transmission season to clear malaria parasites and reduce malaria-related anaemia. This results in a conservative bias of our estimate, although we suggest that combining some activities would plausibly result lower costs. For example, training costs and opportunity costs would be reduced if the training session for teachers occurred only once, and the combined length decreased by a full day. The second point to consider is how these costs might differ under conditions of programme adoption or replication by government agencies, both within this context and in other comparable settings. Costs were split between start up costs, equal to 38% of the total, and operational costs (post start-up) that represent 62% of the intervention. The breakdown suggests that if the programme were to be repeated in the area with the same teachers, there would be cost saving in planning and training activities in subsequent years. The impact on costs attributable to implementation by an INGO, which supported all planning, management and logistical expertise, must be considered in comparison with other implementation bodies. The level of remuneration and the number of staff deployed for the intervention might not be affordable by other actors with fewer resources. Similarly, one of the factors that made training activities expensive was the payment of per diem and transport allowance to the participants. High level of training attendance is crucial for the implementation of the intervention and financial incentives are important in context of limited resources. However, one should consider if the rate set for the allowances was appropriate compared to teachers’ salary. In this study, teachers’ daily salary was 9 USD, while daily per diem was 11.75 USD. Finally, differences within the country are important to consider should the program be rolled out in other regions. Heterogeneities in factors such as accessibility by road, distance between schools, and region size will impact transport and logistical costs. In terms of human resources, excluding Kidal region, the ratio between teachers and children is fairly consistent throughout the country, with a national average of 1:50 [26]. This is larger than the ratio in the study (1:36), highlighting another important area of potential cost-savings given that teachers’ salaries accounted for 15% of total costs reported here. These are among the details warranting further consideration should the government of Mali choose to integrate this intervention into a national strategy. Finally, given that the policy of universal LLIN distribution is now common practice in the Sahel region, removal of the LLIN component from the school program represents a likely scenario for governments in the region. Thus, when considering the replication of a similar school-based intervention in a context with this policy, the LLIN component should be re-evaluated. If removed, our data show that cost per child would decrease to 4.89 USD. This effectively halves the costs of intervention; and is a potentially important finding for policymakers considering complementary strategies to address the burden of malaria among school-aged children.

Additional factors need also be considered in extrapolating these results to other settings. The external validity of these findings may be dependent on factors affecting both the costs and likely effectiveness of this approach. These include, but are not limited to, the dispersion of schools throughout a region, school enrolment rates and regularity of attendance, urban versus rural settings, the seasonality of malaria transmission, and the relative burden of malaria, which was high in this community.

The delivery strategy adopted has a number of potential merits. A major advantage of school-based interventions is availability of personnel, which would be difficult to achieve for programmes requiring health staff in remote settings. The intervention also achieved very high coverage, amongst children enrolled in school. This represents a key strength of the school-based approach in rural, low-resource settings. However, it is important to note the potential negative impacts of relying on school infrastructure to deliver health programmes. A more detailed understanding of the opportunity costs of taking teachers’ and schoolchildrens’ time and re-allocating it to health activities, particularly in settings whose education system already produces poor results, is needed.

Finally, we consider how robust these results are. Sensitivity analyses showed that variation in LLIN price, reduction in salaries for those activities performed by STC personnel, and levels of wastage could all have a substantial impact on cost. All of these aspects can be influenced by the capacity of the actor managing the intervention. A reduced LLIN unit price might be obtained by linking the procurement for this intervention to other national campaigns, and a national authority might be in a better position to do so. In terms of wastage, the intervention showed very low rates that could be attributed to good organization and vigilant management.

### Comparison with previous studies

Cost analyses of combined malaria programmes are limited [11] and, to our knowledge, there are no cost analyses of a similar school-based intervention to allow a direct comparison. Additionally, comparisons are often restricted because of different characteristics of the intervention, different grouping of costs in category and activity, or lack of details about inputs included [21]. Although elements for comparison are limited, high training costs appear to be a distinctive feature of the Mali study. We were unable to identify a cost analysis for comparison concerning school-based LLIN distribution, since current policies typically target children under 5 years and rely on other delivery strategies [27], predominantly door-to-door campaign [21]. According to a review by White et al. [11], the median economic cost per ITN distributed was estimated in USD 4.15, with a range of USD 2.97-10.05; nets and insecticide accounted for 63% of total costs. The cost estimated in our study was 5.53 USD, which falls within this range. Results from an integrated campaign in Togo indicated 4.41 USD per LLIN distributed, and that costs saving were generated from the combined delivery of measles vaccination and LLIN distribution [28]. Finally, cost analysis in Malawi reported that key driver was the cost of the net [29], consistent with our findings in this study.

The IPCs component can be compared with other school-based programmes of drug delivery. In absolute terms, cost per child can be relatively low: examples of deworming interventions administering one drug cost between 0.03 and 0.71 USD [30], and 1.02 USD using two drugs [31]. Drugs usually represent the major cost driver. In an anthelmintic treatment conducted in Tanzania [31], drugs accounted for more than 70% of the total costs as opposed to training which comprised only 10%. Conversely in this study, cost of IPCs was highly determined by personnel costs (56%) and drugs account for just 35%. A more direct comparison can be done with study of intermittent preventive therapy (IPT) conducted among children aged 5-18 in western Kenya [10]. In absolute terms, cost per child was estimated to 1.88 USD, with personnel being the key cost driver (39.6%) followed by drug costs. Although there is a difference in the type of drug administered (SP and amodiaquine in Kenya, versus SP and AS in Mali), this comparison confirms that human resource represents the most relevant cost category to consider. These findings are also consistent with another school based malaria trial in Kenya involving intermittent screening and treatment [13]. Cost comparison was also made with other IPT studies, though they targeted children under 5 only. A study of community-based IPT delivery found that supervision, drug delivery, and training to each account for roughly one-third of costs [16]. Likewise, in a simulation of IPT delivered by community health workers, supervision and drug dispensing-supply were key activities, both accounting for approximately one third of the total, followed by training, representing 20% of the costs [32]. In our study, supervision, which was included in the activity ‘planning and management’, accounted for 7%, drug dispensing and supply for 45%, and training for 38%. This comparison might indicate that IPCs delivery through teachers requires fewer resources for supervision. Finally, a systematic review on costs of malaria interventions indicates personnel and training as the major cost components [11], similarly to this study.

### Limitations

Costs evaluation are challenging because each ingredient generally has a large variability, which could have an impact on the total cost. Providing a probabilistic sensitivity analysis using e.g. Monte Carlo simulation was beyond the scope of our analysis, but represents an avenue for future research. The accuracy of estimation of teachers’ time was limited by inconsistencies in data collection. The activity ‘lessons for children’ time was reported using different units of time (minutes versus fraction of day), and in estimating teachers’ time for administering IPCs, complete forms were available only for a sample of schools. Nevertheless, together these two activities account for only 3% of total costs, and thus are unlikely to have a major impact on our results. An important limitation of this study is that data were collected from a controlled trial rather than during routine intervention, introducing the risk of cost overestimation. The level of rigor needed to monitor scientific trials is generally greater than routine programmes, and these differences may translate directly to greater costs. Likewise, involvement of an international NGO may also be associated with higher costs, for example, the cost of STC personnel and of teachers, as the ratio of children versus teachers in the study is lower than the national average. Further data could be provided by a pilot implementation of the same intervention through local authorities, to investigate possible reduction of costs under routine conditions. Finally, a prerequisite for the implementation of such a programme is a setting of peace and stability, where children can regularly attend school for the entire course of the year, as was the case during the trial period. Conflict, political instability, and poor governance can all potentially undermine effective implementation and increase wastage, ultimately affecting costs and cost-effectiveness.

## Conclusions

Improving child health remains a policy concern in Sub-Saharan Africa and an important objective for the global health community, demanding both innovative and feasible approaches. This study has provided a cost analysis of a comprehensive school-based malaria intervention, in a rural setting with highly seasonal transmission. As in other school-based interventions, teachers represented the key human resources in the actual implementation of the intervention activities, and were able to achieve a high level of coverage. Human resources were estimated as the key cost drivers. Training was the activity that absorbed the largest portion of resources in the intervention as delivered; followed by cost of purchasing LLINs. From a provider perspective, evaluating the total expenditure of 10.38 USD (actual intervention) or 4.85 USD (universal LLIN scenario) per child as an affordable strategy to combat malaria would have to consider the effectiveness, including the health gain and the effects on school outcome of the intervention. Clarke et al. [17] found strong evidence for an impact of the intervention on malaria infection, anaemia, and haemoglobin concentration, as well as significant improvements in cognitive function, and this effect was found to endure throughout the low-transmission period. Taken together with the epidemiological findings, the current cost analysis provides impetus for the consideration of comprehensive school-based malaria control programs to target the burden of malaria in school-aged children in the Sahel region.

## Additional files

Additional file 1: Unit Costs. A list of ingredient unit costs and relevant data collected for this evaluation. Description of data: based on ingredient approach, the table presents resources necessary to carry out the intervention. Items are listed, measured, valued and grouped in cost categories. (DOCX 23 kb)

Additional file 2: Detailed financial and economic costs of comprehensive’ malaria intervention strategy for use in schools in areas of seasonal transmission: Adapted version for future scale-up. Description of data: the table provides financial and economic costs for the adapted intervention, considering distribution of 1 LLIN instead of 2 LLINs per child (actual implementation. Costs are split by cost category and by activities. (DOCX 20 kb)

## Abbreviations

ACT: Artemisin-based Combination Therapy
AQ: Amodiaquine
AS: Artesunate
CDA: Community Development Agent
DTC: Directeur Technicien de Santé Communautaire
INGO: International Non-Governmental Organisation
INRSP: Institute National de Recherche en Santé Publique
IPCs: Intermittent Parasite Clearance in schools
ITN: Insecticide-Treated Net
LLIN: Long lasting insecticidal net
LSHTM: London School of Hygiene and Tropical Medicine
PNLP: Programme National de Lutte contre le Paludisme
SP: Sulfadoxine Pyrimethamine
STC: Save The Children
USD: United States Dollars
WHO: World Health Organization
XOF: West African Francs (Francs de la Communauté Financière Africaine)
