# Supplementary material for: Cost analysis of a school-based comprehensive malaria program in primary schools in Sikasso region, Mali
Source: BMC Public Health. 2017 Jun 12;17:572. doi: 10.1186/s12889-017-4490-6 (PMC5469144; doi:10.1186/s12889-017-4490-6)
Supplement: Supplementary file 1 — Unit Costs. A list of ingredient unit costs and relevant data collected for this evaluation. Description of data: based on ingredient approach, the table presents resources necessary to carry out the intervention. Items are listed, measured, valued and grouped in cost categories. (DOCX 23 kb) [file 12889_2017_4490_MOESM1_ESM.docx]

**Additional File 1:** Unit Costs. A list of ingredient unit costs and relevant data collected for this evaluation.

| **Cost category** | | **Item** | | | **Source** | | | **Unit** | | | **Unit cost (in XOF)** | | |  |  |
| --- | --- | --- | --- | --- | --- | --- | --- | --- | --- | --- | --- | --- | --- | --- | --- |
| **Human resources** | Salary supervisor/coordinator 1 (STC staff) | | STC | | | | day | | | 32088 | | |  |  |  |
|  | Salary supervisor/coordinator 2 (STC staff) | | | STC | | | day | | | 32261 | | |  |  |  |
|  | School Health and Nutrition Program Coordinator (STC) | | | STC | | | day | | | 23169 | | |  |  |  |
|  | Salary trainer 1 (STC staff) School Health and Nutrition Manager (middle position), | | | STC | | | day | | | 32088 | | |  |  |  |
|  | Salary trainer 2 (STC staff) Sikasso Impact Area Coordinator (senior position) | | | STC | | | day | | | 45043 | | |  |  |  |
|  | Salary trainer 3 (STC staff) CDAs (field workers – junior position). | | | STC | | | day | | | 14561 | | |  |  |  |
|  | Salary trainer 4 (STC staff) CDAs (field workers – junior position). | | | STC | | | day | | | 14561 | | |  |  |  |
|  | Salary logistician/stock management (STC staff) | | | STC | | | day | | | 9454 | | |  |  |  |
|  | Salary admin/finance (STC staff) | | | STC | | | day | | | 35973 | | |  |  |  |
|  | Salary driver (STC staff) | | | STC | | | day | | | 9631 | | |  |  |  |
|  | Trainer per Diem | | | STC | | | day | | | 15000 | | |  |  |  |
|  | Teacher per Diem day 1-2 | | | STC | | | day | | | 12000 | | |  |  |  |
|  | Teacher per Diem day 3 (half day) | | | STC | | | day | | | 6000 | | |  |  |  |
|  | DTC Per Diem | | | STC | | | day | | | 12000 | | |  |  |  |
|  | STC Supervisor transport allowance | | | STC | | | person | | | 82960 | | |  |  |  |
|  | Teachers transport allowance | | | STC | | | person | | | 5000 | | |  |  |  |
|  | Transport allowance DTC | | | STC | | | person | | | 5000 | | |  |  |  |
|  | Accommodation allowance Teachers day 1,2 | | | STC | | | day | | | 2500 | | |  |  |  |
|  | Accommodation allowance Teachers day 3 | | | STC | | | day | | | 2500 | | |  |  |  |
|  | Accommodation allowance DTC | | | STC | | | day | | | 2500 | | |  |  |  |
|  | Per diem trainer (STC) | | | STC | | | day | | | 6000 | | |  |  |  |
|  | Per diem driver (STC) | | | STC | | | day | | | 6000 | | |  |  |  |
|  | Per diem health officer (1) | | | STC | | | day | | | 6000 | | |  |  |  |
|  | Per diem health officer (2) | | | STC | | | day | | | 4500 | | |  |  |  |
|  | Teacher allowance for out-reaching | | | Per test for adapted intervention | | | per child | | | 300 | | |  |  |  |
| **Training Materials** | Teachers' manual | | | STC | | | item | | | 2110 | | |  |  |  |
|  | Trainers' manual | | | STC | | | item | | | 1500 | | |  |  |  |
|  | Photocopies in house | | | STC | | | item | | | 3000 | | |  |  |  |
|  | Notepads (100 pages) | | | STC | | | item | | | 135 | | |  |  |  |
|  | Notepads(200 pages) | | | STC | | | item | | | 280 | | |  |  |  |
|  | Pens | | | STC | | | item | | | 65 | | |  |  |  |
|  | Pre-tests copies | | | STC | | | item | | | 25 | | |  |  |  |
|  | Forms for data collection | | | STC | | | item | | | 25 | | |  |  |  |
|  | Guideline for IPCs treatment | | | STC | | | item | | | 25 | | |  |  |  |
|  | Contact sheets | | | STC | | | item | | | 25 | | |  |  |  |
|  | Markers | | | STC | | | item | | | 1600 | | |  |  |  |
|  | Flipchart | | | STC | | | item | | | 3650 | | |  |  |  |
|  | Net used for training purpose | | | STC | | | item | | | 2500 | | |  |  |  |
|  | Refreshment day 1-2 | | | STC | | | centre/day | | | 60000 | | |  |  |  |
|  | Refreshment day 3 | | | STC | | | centre/day | | | 24000 | | |  |  |  |
| **Logistics and Transport** | Vehicle Toyota Land Cruiser (20.250.000 XOF - 10ys life span) | | | STC | | | day | | | 7788 | | |  |  |  |
|  | Other vehicle costs (insurance, maintenance, inspection) | | | STC | | | day | | | 3496 | | |  |  |  |
|  | Fuel for vehicles (20 litres of fuel per 100 km= 5km/lit) / 715 XOF/lit | | | STC | | | fuel per km | | | 143 | | |  |  |  |
|  | Fuel for motorbike (5 litres of fuel per 100 km = 20km/lit) - 630 XOF/lit | | | STC | | | fuel per km | | | 31.5 | | |  |  |  |
|  | Transport of drugs from Bamako to Sikasso 380km (610 xof/1 litre of petrol, 20 litres of fuel for 100 kilometres=5km/lit) | | | STC | | | fuel per km | | | 122 | | |  |  |  |
|  | Rent storage unit (total storage area 9m50 by 7m20 = 68.4m2; space occupied by net = 4m2, equal to 6% of total) - for 3.5 months | | | STC | | | month | | | 65000 | | |  |  |  |
|  | Customs cost for importing drugs (606.313 XOF per 9.500 tablet) | | | STC | | | tablet | | | 63.82 | | |  |  |  |
|  | Communication: pre-paid cards during training | | | STC | | | card | | | 5000 | | |  |  |  |
|  | Communication cost phone Sikasso | | | STC | | | day | | | 4475 | | |  |  |  |
|  | Communication cost internet Sikasso | | | STC | | | day | | | 7845 | | |  |  |  |
|  | Communication: pre-paid cards for CDA | | | STC | | | card | | | 2500 | | |  |  |  |
| **Intervention items** | LLIN (2.500 XOF each, 3 years life span) | | | STC | | | net/year | | | 833.33 | | |  |  |  |
|  | Wastage factor LLIN (lost 240 nets out of 16.000) | | | STC | | | % | | | 0.015 | | |  |  |  |
|  | Drugs (ARTECOSPE-specification 6+3) quantity 26%, unit price USD 1,40 | | | STC | | | tablet | | | 690.19 | | |  |  |  |
|  | Drugs (ARTECOSPE-specification 6+2) quantity 74%, unit price USD 0,85 | | | STC | | | tablet | | | 419.04 | | |  |  |  |
|  | Wastage factor drugs | | | NA | | | % | | | 0 | | |  |  |  |
| STC: Save The Children  DTC: Directeur Technicien de Santé Communautaire  CDA: Community Development Agent  LLIN: Long Lasting Insecticidal Net  IPC: Intermittent Parasite Clearance | | | | | |  | | |  | | |  | | |  |
