# Supplementary material for: Cost analysis of a school-based comprehensive malaria program in primary schools in Sikasso region, Mali
Source: BMC Public Health. 2017 Jun 12;17:572. doi: 10.1186/s12889-017-4490-6 (PMC5469144; doi:10.1186/s12889-017-4490-6)
Supplement: Supplementary file 2 — Detailed financial and economic costs of comprehensive’ malaria intervention strategy for use in schools in areas of seasonal transmission: Adapted version for future scale-up. Description of data: the table provides financial and economic costs for the adapted intervention, considering distribution of 1 LLIN instead of 2 LLINs per child (actual implementation. Costs are split by cost category and by activities. (DOCX 20 kb) [file 12889_2017_4490_MOESM2_ESM.docx]

**Additional file 2.** Detailed financial and economic costs of comprehensive ’ malaria intervention strategy for use in schools in areas of seasonal transmission: Adapted version for future scale-up

|  | **Adapted Intervention 1^1^** | | | | | |
| --- | --- | --- | --- | --- | --- | --- |
|  | Financial cost (in XOF) | Financial cost (in USD) | Cost profile | Economic cost (in XOF) | Economic cost (in USD) | Cost profile |
|  | By Cost category | | | | | |
| **Start-up Costs** |  |  |  |  |  |  |
| Human Resources | 9,012,403 | 17,654 | 41% | 11,635,846 | 22,793 | 41% |
| Materials | 1,321,640 | 2,589 | 6% | 1,321,640 | 2,589 | 5% |
| Logistics and transport | 20,000 | 39 | 0.1% | 20,000 | 39 | 0.1% |
|  |  |  |  |  |  |  |
| **Operational Costs (post start-up)** | |  |  |  |  |  |
| Human resources | 2,404,975 | 4,711 | 11% | 5,430,239 | 10,637 | 19% |
| Logistics and transport | 916,117 | 1,795 | 4% | 1,057,589 | 2,072 | 4% |
| Intervention items |  |  |  |  |  |  |
| LLINs^1^ | 5,424,329 | 10,626 | 24% | 5,753,018 | 11,269 | 20% |
| Drugs | 3,139,427 | 6,150 | 14% | 3,139,427 | 6,150 | 11% |
|  |  |  |  |  |  |  |
|  | By Activity | | | | | |
| Planning and management | 2,149,871 | 4,211 | 10% | 2,461,362 | 4,822 | 9% |
| Developing teaching material | 343,203 | 672 | 2% | 412,424 | 808 | 1% |
| Community sensitization | 32,427 | 64 | 0.1% | 1,368,375 | 2,680 | 5% |
| Training | 9,416,960 | 18,447 | 42% | 12,091,183 | 23,685 | 43% |
| Lessons for children | 0 | - | 0% | 584,477 | 1,145 | 2% |
| LLIN purchase, transport and storage | 6,122,702 | 11,994 | 28% | 6,462,127 | 12,659 | 23% |
| LLIN distribution | 367,640 | 720 | 2% | 800,081 | 1,567 | 3% |
| Drugs purchase, transport and storage | 3,665,643 | 7,181 | 16% | 3,835,798 | 7,514 | 14% |
| Drugs dispensing | 140,445 | 275 | 1% | 341,933 | 670 | 1% |
|  |  |  |  |  |  |  |
| **Total** | 22,238,891 | 43,563 | 100% | 28,357,759 | 55,550 | 100% |
| ^1^ Assumes 1 LLIN distributed to each schoolchild each year, compared to 2 LLINs per child in actual implementation. However costs could potentially be reduced further if LLIN distribution to all pupils in schools is only carried out every 3 years, supplemented with annual distribution in intervening years limited to newly-enrolled children in grade 1 only | | | | | | |
